# 

**Published:** 1888-04-28

**Authors:** 


### HOSPITAL SATURDAY MOVEMENT.

THE following is a syllabus of the paper to be read by Mr. A. W. Webster (Chairman of Workmen's Governors Sunderland Infirmary), at the Memorial Hall, Farringdon Street, on Monday, April 30th, at eight p.m., to the members and friends of the Hospital Saturday Movement. We publish it beforehand in order that those who purpose to attend may be the better prepared to take an intelligent part in the discussion. The title of the paper is "How to extend the usefulness of the Hospital Saturday Movement, with special reference to workmen's subscriptions."

SYLLABUS.—Workmen's Organisation for Hospital Support in Sunderland—Workmen's Subscriptions in other Towns—Resolution to Adopt the Penny-a-week Scheme for London—Proposed Pamphlet to London Workmen (Present Hospital Saturday Movement—Proposed New Scheme—How the System of Regular Contributions was Started in Sunderland—Introduction of the Penny-a-week Scheme to London)—Alterations of Constitution—Organisation—How to Circulate the Pamphlet and Make it Pay the Cost of Publication—Employers' Interest in the Work—Meetings Conducted by Local Committees—Supplementary Work (Occasional Schemes—Extension of Collections on Hospital Saturday—Proposed Children's Fund—Additional Schemes for Ladies—Outlying Districts)—System of Gathering Accumulated Collections.

### QUESTIONS AND ANSWERS.

COULD anyone kindly tell a country Vicar of a Home where a boy (15) subject to occasional epileptic fits, could be received, where he would meet with medical care, and where he could be taught a trade. A small weekly payment would be given. Parents very respectable, but poor.

Rugby. C. H. D.

### Special Notice to Newsagents, Advertisers, and the Trade.

**CHANGE OF OFFICE AND PUBLISHER.**

Notice is hereby given that the Office of The Hospital will henceforth be at 38, Old Jewry (Cheap·ide end), E.C., to which address all communications should be sent. Mr. A. Doig has been appointed Advertising Agent, and Mr. J. H. S. Hanning, Manager. All Cheques and Post Office Orders should be made payable to J. H. S. Hanning, 38, Old Jewry, E.C. crossed Lloyds, Barnetts, and Bosanquets Bank (Limited).

### To Secretaries and Matrons.

The Editor will be glad to have a copy of the Regulations, and also of every Report issued from the Press of Asylums, Hospitals, Convalescent and Nursing Institutions, as well as those of other Charities, so soon as they are published, and an early copy in duplicate of all appeals for funds. Notices of Annual and other Meetings, Festival Dinners, etc., will be welcomed. All such communications should be addressed to *The Editor, The Lodge, Porchester Square, London, W.* Any superintendent, secretary, matron, or other chief official who will regularly send such documents and information will be registered to receive free of cost a cover for binding The Hospital in half-yearly volumes on sending name and address to the publisher.

By the law of diffusion of gases, at work by day and night, in sunlight and in dark, in summer and in winter, all noxious gases and vapours which are allowed to pass into the open air are quickly diffused throughout the atmosphere. Winds waft the air to and fro on the surface of the earth. Rain showers tend to purify the atmosphere by washing down the impurities to fertilize the soil. Electric storms assist in clearing the air of noxious principles, and plants are ready to absorb the deadly carbonic acid, and give us back pure life-inspiriting oxygen in its stead. Our part of the purifying work is very simple. All we need do is to cease imprisoning the polluted air within our homes, and put forth our hand to liberate the deadly and poisoned air, and the God-ordained natural forces and agencies then complete the mighty task.

### LONDON CHARITIES: QUINN'S AND GRAHAM'S BEQUESTS.

IT has become more and more the fashion of late years for the wealthy to leave their money to be distributed after their death at the discretion of their executors. In the case of the late Mr. Graham, who left upwards of £100,000, which was distributed in this way ten years' ago—*i.e.*, at the end of 1877—the acknowledgments occupied three advertisement columns of the *Times*. The hospitals and kindred charities received just half the amount to be distributed, or £50,000; £8,500 was given to colleges and schools; £7,000 and £7,250 respectively to voluntary homes and orphanages; £6,750 to the deaf-and-dumb charities; and the balance was distributed in smaller sums over the whole charitable field. Nothing was, however, given to churches and chapels, or to Bible and Book societies. This year the late Mr. Henry Quinn, of 328, Mansion House Chambers, and 11, Queen Victoria Street, bequeathed the munificent sum of £50,000 for distribution among the metropolitan charities. The executor, Mr. W. M. Hepper, who was entrusted with discretionary powers under the will, having seen our request for full particulars, has courteously forwarded to us a list of the charities benefited, and the amounts apportioned to each. It will be noticed that the largest amount—£5,000—has been given to the Great Northern Central Hospital in the Holloway Road. That hospital is now erecting a new building at a cost of £50,000; and the £5,000 received under Mr. Quinn's bequest has been appropriated to the endowment of a ward, to be named after the testator the *Henry Quinn Ward*. Mr. W. M. Hepper had a difficult task assigned him, and he is certainly to be congratulated upon the manner in which he has fulfilled it. The hospitals and kindred charities receive rather more than half the money, or £28,400; £6,300 is distributed amongst orphanages and other children's charities; £5,500 is given to voluntary homes and to reformation and preservation agencies; and the only groups which receive nothing are—(*a*) Bible, book, and tract societies, (*b*) missions, and (*c*) social improvement institutions. We will now give the list of charities receiving grants from the Quinn's bequest in detail, as it cannot fail to be of the greatest interest to many beside those interested in the particular charities concerned. The classification adopted will make it more easy to compare and understand the figures:

### MR. QUINN'S LEGACY, 1888.

**Class I.—Sickness**

**General Hospitals**—Charing Cross, £1,200; Great Northern Central, £5,000; Guy's, £2,500; King's College, £1,200; London, £1,500; Metropolitan Free, £400; Middlesex, £1,200; North-West London, £100; Richmond, £1,000; St George's, £1,000; St. Mary's, £1,000; Seamen's, £500; University, £1,000; West London, £300; Westminster, £800 £18,700

**Hospitals for Women and Children**—East London Hospital for Children, £300; Evelina Hospital for Children, £500; Hospital for Women, Soho Square, £500; Hospital for Children, Great Ormond Street, £600; North-Eastern Hospital for Children, £500; Royal Hospital for Women and Children, £200; Samaritan Free for Women, £500; Victoria Hospital for Children, £400 .. .. .. .. .. .. £3,500

**Special Hospitals**—British Lying-in Hospital, £200; Cancer Hospital, £1,000; City of London Hospital for Diseases of the Chest, £800; Royal Hospital for Diseases of the Chest, £500; Royal National Hospital for Consumption, Ventnor £500; Hampstead Home Hospital, £100; Hospital for Epilepsy and Paralysis, £200; Hospital for Skin Diseases, £200; London Fever Hospital, £600; London Temperance Hospital, £300; National Hospital for Diseases of the Heart, £200; Royal Westminster Ophthalmic Hospital, £300 .. £4,900

**General Dispensaries**—City Dispensary, £200; North-West London Dispensary, £100; Surrey Dispensary, £200.. £500

**Other Agencies**—The North London Nursing Association, £300; Metropolitan Convalescent Home, £300 .. .. £600

**Surgical Appliances**—Provident Surgical Appliances Institution, £200; Surgical Aid Society, £200; City of London Truss Society, £300 .. .. .. .. .. .. £700

**Class II.—Charities for those Permanently Afflicted:** British Asylum for Deaf-and-Dumb Females, £100; Home for Confirmed Invalids, £300; Indigent Blind Visiting Society, £100; London Home for Aged Blind Men and Women, £500; Royal Association for Deaf and Dumb, £300; Royal Normal College for the Blind, £200; Surrey Association for the Blind £200; School for Indigent Blind, £500; London Society for Teaching the Blind, £100.. .. £2,300

**Class III.—Pensions for the Aged, etc.:** Aged Pilgrims' Friend Society, £200; Friend of the Clergy Corporation, £200; United Kingdom Beneficent Association, £1,000 .. .. .. .. .. .. .. .. .. .. £1,400

**Class IV.—Asylums, etc., for the Aged:** Cambridge Asylum for Soldiers' Widows, £200; Royal Alfred Asylum for Aged Merchant Seamen, £500 .. .. £700

**Class V.—Relief of Distress:** Governesses' Benevolent Institution, £1,000; Metropolitan Visiting Relief Association, £500; Scottish Corporation, £200; Society for Widows and Orphans of Medical Men, £500; Railway Benevolent Institution, £1,000; Widows' Fund, £100 .. .. .. .. .. .. .. .. £3,300

**Class VI.—Charities for Orphans and Children:** Asylum for Fatherless Children, Slough, £500; British Orphan Asylum, £500; Boys' Home, Regent's Park, £200; Female Orphan Asylum, £500; Girls' Home, £100; Home for Little Boys, £500; Infant Orphan Asylum, Wanstead, £1,000; London Orphan Asylum £1,000; National Orphan, Home, Ham, £500; Orphan Working School, £500; Royal School for Daughters of Officers in the Army, £200; Stockwell Orphanage, £500; Soldiers' Daughters' Home, Hampstead, £300 .. .. .. .. .. .. .. .. £6,300

**Class VII.—Churches and Chapels:** St. Paul (E) Docks . .. .. .. .. .. .. £200

**Class VIII.—Voluntary Homes:**

**Charities for Reformation and Preservation**—Dalston Refuge, £200; Discharged Prisoners' Aid Society, £500; Industrial Home for Boys, £600; National Refuge, for Homeless and Destitute Children, £1,000; Royal Marine Society, £1,000; Gray's Yard Ragged School, £200; Ragged School Union, £1,000; Field Lane Refuge and Ragged School, £300; National Industrial Home for Crippled Boys, £300; Newport Market Refuge, £200; Refuge for Deserted Mothers, £100; Princess Louise's, Home, £100 .. .. .. .. .. .. .. .. £5,500

**Class IX.—Education;**

**Colleges, Schools, &c.**—Richmond National School .. £100

**Class X.—Protection and Preservation:** Royal Humane Society, £500; Royal National Lifeboat Institution, £500; Society for Rescuing Young Women and Children, £300 .. .. .. .. .. .. .. .. £1,300

Total, £50,000

### MR. GRAHAM'S LEGACY, 1877.

IT is interesting to compare Mr. Hepper's distribution with that of Graham's executors, which latter is here reproduced for purpose of comparison.

| | | | |
|---|---|---|---|
| Class I.—Literary.... | | | — |
| Class II.—2 Missionary | | | £750 |
| Class III.—Church and Chapel Building | | | — |
| Class IV.—9 Affliction (Blind, Deaf and Dumb, and Idiots | | | 6,750 |
| Class V.—56 Sickness, viz: 14 General Hospitals £19,500 | | | |
| Special Hospitals:— | | | |
| 5 Consumption | 6,750 | | |
| 3 Ophthalmic | 1,750 | | |
| 1 Orthopædic | 250 | | |
| 1 Skin | 500 | | |
| 6 Women | 2,750 | | |
| 4 Children | 4,500 | | |
| 2 Lying-in | 750 | | |
| 6 Miscellaneous | 3,500 | | |
| 3 Lock and Fever | 2,000 | | |
| 1 Cancer | 1,000 | | |
| 2 Incurables | 3 000 | | |
| | | £46,250 | |
| 1 General Dispensary | | 500 | |
| 3 Convalescent Homes | | 1,500 | |
| 2 Nursing Institutions | | 750 | |
| 2 Truss and Surgical Aid Societies | | 1,000 | |
| | | | 50,000 |
| Class VI.—7 Annuities—Pensions, &c., for the Aged | | | 2,750 |
| Class VII.—4 Almshouses, Asylums, etc., for ditto | | | 2 750 |
| Class VIII.—6 Distress—General Relief | | | 4,000 |
| Class IX.—13 Voluntary Homes—Ladies, Governesses, Women, Girls, Men, Boys, Infants, etc. | | | 7,250 |
| Class X.—9 Orphans—Asylums, Homes, Schools, etc. | | | 7,000 |
| Class XI.—8 Reformation and Prevention—Industrial, Reformatory, and other Schools, Discharged Prisoners, etc. | | | 4,000 |
| Class XII.—12 Education—Colleges and Schools | | | 8,500 |
| Class XIII.—2 Social Improvement | | | 750 |
| Class XIV.—3 Protection and Preservation | | | 3,500 |
| | | | £98,000 |

### AN AUTOMATIC QUACK!

THE latest form of quack doctor, according to an American newspaper, is an automatic pill-vendor. It resembles the automatic sellers of cigars and sweets now in operation at railway stations. The mechanical quack is supplied with a variety of pills suited to all kinds of cases. The patient puts his coin into the slit over the particular remedy he selects, and out comes the ready-made pill for his use. Orthodox practitioners might take the hint, and insist upon the fee being put into the slit before the advice and medicine are supplied.

### Amusements with Profit for all Ages.

**Rules.**—All answers must be addressed to the Prize Editor, The Hospital, 38, Old Jewry, Cheapside, London, E.C., not later than the *first* post on Thursday next. The full name and address must in all cases be given, but a *nom de plume* may be added for publication. Only one side of the paper must be written on.

### FOR MEN AND WOMEN.

**Rule.**—Competitors can enter for *all* quarterly competitions, but no competitor may take more than *two quarterly prizes* during the year.

**Acrostic Competition.**

Second quarter commenced February 25th, 1888; ends May 19th, 1888.

A PRIZE of ONE GUINEA will be given to the competitor who gains the highest number of marks for best original acrostics during the ensuing quarter. All acrostics sent in for this competition will be credited according to merit, twelve marks being the highest score given for one acrostic.

A PRIZE of HALF-A-GUINEA to be given to the competitor who gains the highest score for solution of acrostics set. *One mark* only will be given to the competitors who solve all the lights of each acrostic.

Exception will be made in the case of *quotation acrostics*, when *two marks* will be given, *one* for the correct solution of all *lights*, and *one* mark for the *source* of the quotations.

This week credit will be given for

**No. 5.—Original Double Acrostic.**

**Results of No. 4 Original Double Acrostic.**

Brownie (12), Patience (10), A. M. E. (9).

(A) THE WORD COMPETITION.

Second Quarterly Competition commenced February 4th, 1888; ends April 28th, 1888.

Three prizes of one guinea, 15*s.* and 10*s.* 6*d.* will be given each quarter to the three competitors who obtain the highest number of marks: (1) by making the largest number of words derived from the word or phrase set for anatomical dissection; or (2) for the best answers to (B); or (3) by (1) and (2) combined.

We shall give the newspapers and periodicals for dissection during this quarter, that for this, the *thirteenth* week of the quarter, being

" PASTIME."

Observe.—Proper names, abbreviations, foreign words, words of less than four letters, and repetitions are barred; plurals, and past and present participles, of verbs are allowed. Nuttall's *Standard* dictionary only to be used.

N.B.—All words sent in must be arranged *alphabetically*, with correct total affixed.

**Results of Eleventh Week.**

Patience (76), Sym (76,) Ellice (70), Lauriston (67), Lightowlers (67), E. E. (63), Dunce (62), Gretta (61), Brisbane (61), Butterfly (61), Ecila (59), Oliver (53).

(B) THE LITERARY COMPETITION.

Some people hate puzzles of all kinds, including word competitions, but they are fond of correspondence, and many sisters and mothers are continually receiving and sending letters to and from members of the family who are abroad, in India or the Colonies. The constant coming and going between England and other portions of the British Empire make it a matter of interest and importance to know something about the life, the climate, the amusements, the adventures, the clothing, and the surroundings of those who seek a livelihood beyond the seas. We shall therefore try to find space for the publication of selected letters from abroad, which may be sent by any of our readers who have friends and relations in foreign lands. We shall give marks for these letters as well as for the word competitions, which will count equally for these prizes, so that anyone may enter for both or either, the prizes to be given to those who get the highest number of marks.

### THE CHILDREN'S CORNER.

PRIZES.
FOR MOST CORRECT ANSWERS TO PUZZLES.
This Commenced October 1st, 1887.
One Pound a Month.

**Puzzles—Fourth Week.**

No. 13. Double Acrostic.

Two monsters of the deep we be—
The wealth, yet terror of the sea.

1. *He*, with the carpenter of oft-told tale,
Misled by guile his kindred to their bale.
2. With brother butterfly in summer bowers
I dart and drop upon the fairest flowers.
3. Beware, Oh traveller! my dread embrace;
My tribe's the deadliest foe of human race.
4. My hold's tenacious and my hue is red,
And men do seek me most when I'm in bed.
5. The monarch of the forest glade,
From me is many a trophy made.

No. 14. Arithmetical Puzzle.—A gentleman meeting a certain number of schoolboys; and, being desirous to distribute among them all the money he had with him, finds that if he gave them sixpence each, he would have 2s. too little; but by giving to each fourpence he would have 2s. 8d. over. How many schoolboys were there, and how much money had the gentleman in his pocket?

No. 15. Charade.

My first is irrational,
My second rational,
My third mechanical,
And my whole scientific.

No. 16. Whom does the following quotation from Shakespeare describe

" His nature is too noble for the world:
He would not flatter Neptune for his trident,
Or Jove for his power to thunder. His heart's his mouth:
What his breast forges, that his tongue must vent;
And being angry, does forget that ever
He heard the name of death."

**Results of Second Week.**

No. 5. The four poets transposed are:

Shelley—" Queen Mab."
Tennyson—" Idylls of the King."
Longfellow—" Evangeline."
Wordsworth—" The Excursion."

No. 6. Square Word.

H A R P
A L O E
R O P E
P E E L

No. 7. Double Acrostic.

R oll O
A die U
M ohamme D
I vanho E
L angto N
L oyol A
I ndia Rubbe R
E nglan D
S toneheng E

No. 8. Charade. Ire, land = Ireland.

*All right.*—A. C. K., Good Old Killaloe, Bunny, Dame Durden, M. M. S., Scrooge, Gem, Hercules, Nunkey, Spider, Mount Edgecumbe. *Three right.*—McCulloch, Plummy Fluff.

### The Student's Column.

### UNIVERSITY OF EDINBURGH.
### PROFESSIONAL EXAMINATIONS.

The following is the official list of candidates who passed the first professional examination in March:—

J. C. Addingley, A. J. Anderson, F. T. Auden, F. R. K. Ball, J. T. Balmford, William Bannerman, H. M. Benson, A. F. M. Berkeley, George Bidie, T. H. Bishop, M. R. Bow, D. C. Bremner, B. W. Broad, J. H. Brown, Alexander Brownlie (with distinction), P. G. van der Byl, John Cadwaladr-Williams, W. M. Cairns, D. K. Cameron, H. A. Clark, G. E. Clemens, R. P. Cockburn, Arthur Dennison, J. A. Dick, T. G. Dickson, W. G. Donald, Henry Duckworth, John Elliot, J. H. Ewart, R. W. Fell, F. D. Fisher, William Forsyth, A. M. Fraser, J. H. Furniss, R. A. Fryer, H. C. Garth, S. B. Gay, T. G. Goldie-Scot, Edmund Gonin, William Gordon, A. D. M. Grant, E. C. Greaves, T. B. Green, George Guild, F. E. Gunter, H. B. Hall (with distinction), H. P. Hannay, W. A. Hardiker, W. B. Harry, Edgar Hawkins, B.A., W. P. Hay, J. V. Hulme, G. P. Humphrey, C. P. Hurst, T. W. Iddon, O. H. Izard, A. K. Jarratt, John Joakim, J. H. Johnston, T. O. Jones, J. M. Kay, Forrester Kennedy, W. C. C. Kirkwood, J. R. P. Lambert, R. E. Little, William Macalpine, H. M. Macgill, Wakefield Macgill, G. A. I. Mackay, Murdo Mackay, W. H. Mackay, J. A. Mackenzie, W. H. Mackinlay, A. J. Mackintosh, J. H. Mackintosh, John M'Laren, J. D. Maclean, Gustav Mann, James Marsh, C. B. Martin, J. L. Martin, J. A. Menzies, A. S. Miller, Alexander Mitchell, Ahmed Mirza, R. U. Moffat, J. R. Monnier, C. E. Moody, E. C. Moore, H. B. T. Morgan, G. C. Munnik, J. J. M. Muir, H. A. E. Noble, E. R. Parry, F. R. Patterson, S. P. Peart, James Philip, P. H. du Plessis, J. C. Potter, G. H. Prance, R. L. Price, E. C. Quertier, J. L. Reed, T. H. Reed, J. M. Renton, M.A., Stanley Riseley, Edward Rowan, J. L. Rubidge, Andrew Rutherford, Ahmed Ruza, J. B. Shaw, Theodore Shennan, F. M. T. Skae, E. W. Slayter, J. B. Smith, P. C. C. Smith, E. M. Steven, Hugh Steven, Robert Strachan, John Sutherland, F. A. Symons, E. S. Towert, A. H. Walker, Alban Ward, J. F. Watson, John Webster, Reginald Williams, L. U. Young, R. J. C. Young.

The following is the official list of candidates who have passed the second professional examination during this month:

C. A. Anderson, C. M. Anderson, R. H. Beardsley, G. E. A. G. Becker, J. D. Broadfoot, John Brown Lester, L. C. Bruce, W. A. Bryant, R. D. Buchanan, Barend Burger, W. H. Bunting, Thomas Burns, W. C. Burns, A. W. Campbell, D. A. Carruthers, A. W. Carter (with distinction), Frederick Clarke, A. G. Coles, J. W. Crerar (with distinction), G. M. Cullen, James Currie, Henri Dardenne, F. A. Day, J. R. Dickson, Herbert Doble, C. C. Douglas (with distinction), J. J. Douglas, J. W. Dowden, E. H. Duncan, M.A. (with distinction), E. E. Dyer, Samuel Elliot, William Everett, H. C. Evison, J. A. Ewan, Samuel Ferguson, Denson Gibb, W. S. Gibb, W. S. Gibson, Colin Gordon (with distinction), D. H. Graves, J. D. Gray, Guy Grindlay, A. O. Harkness, J. W. Hart, M. L. Hewat, L. W. Hignett, A. C. Houston, H. C. Hudson, W. H. Hughes, David Huskie, H. M. Inglis, H. de D. Kunz (M.A.), James Lackie (with distinction), C. B. Lawson, A. B. Lazarus, William Leask, J. W. Leitch, Henry Leschen, T. H. Littlejohn, F. A. L. Lockhart, G. V. Lockett, James Luke, R. P. Mackenzie, C. C. Macknight, John M'Laren, Alexander M'Lean (with distinction), E. J. Maclean, C. G. Macleod, A. W. Messer, E. A. Milner, James Monteith, A. W. Mortlock, H. T. Mursell, William Oliver, W. M. Parham, E. G. Pilgrim (with distinction), George Porter, F. A. G. Purchas, E. W. Rahn, W. H. Ralphs, A. R. Ritchie, T. M. Ritchie, A. G. Robertson, J. H. Robertson, C. J. Robson-Scott, R. D. Rudolf, J. A. Scott, L. S. Senhouse, W. J. Shaw, J. L. Smith, Walter Smithies, E. G. B. Starkie, K. T. Stewart, A. N. J. Story, Benjamin Sweeten, Alexander Thomson, A. C. Thomson, W. E. F. Thomson, George Thyne, J. A. Thyne (with distinction), A. J. Wallace, G. G. Watson, Herbert Watthews, David Westwood, P. Carr White, A. W. Wilcox, George Wilkinson (with distinction), John Wilson (M.A.)

